# Implantation of neural stem cells embedded in hyaluronic acid and collagen composite conduit promotes regeneration in a rabbit facial nerve injury model

**DOI:** 10.1186/1479-5876-6-67

**Published:** 2008-11-05

**Authors:** Han Zhang, Yue Teng Wei, Kam Sze Tsang, Chong Ran Sun, Jin Li, Hua Huang, Fu Zhai Cui, Yi Hua An

**Affiliations:** 1 Beijing Neurosurgical Institute, Capital Medical University, Beijing, PR China; 2 Department of Materials Science and Engineering, Tsinghua University, Beijing, PR China; 3 Department of Anatomical and Cellular Pathology, Chinese University of Hong Kong, Hong Kong, PR China; 4 Li Ka Shing Institute of Health Sciences, Chinese University of Hong Kong, Hong Kong, PR China; 5 Department of Neurosurgery, Second Affiliated Hospital of Zhejiang University Medical College, Hangzhou, PR China

## Abstract

The implantation of neural stem cells (NSCs) in artificial scaffolds for peripheral nerve injuries draws much attention. NSCs were *ex-vivo *expanded in hyaluronic acid (HA)-collagen composite with neurotrophin-3, and BrdU-labeled NSCs conduit was implanted onto the ends of the transected facial nerve of rabbits. Electromyography demonstrated a progressive decrease of current threshold and increase of voltage amplitude in de-innervated rabbits after implantation for one, four, eight and 12 weeks compared to readouts derived from animals prior to nerve transection. The most remarkable improvement, observed using Electrophysiology, was of de-innervated rabbits implanted with NSCs conduit as opposed to de-innervated counterparts with and without the implantation of HA-collagen, NSCs and HA-collagen, and HA-collagen and neurotrophin-3. Histological examination displayed no nerve fiber in tissue sections of de-innervated rabbits. The arrangement and S-100 immunoreactivity of nerve fibers in the tissue sections of normal rabbits and injured rabbits after implantation of NSCs scaffold for 12 weeks were similar, whereas disorderly arranged minifascicles of various sizes were noted in the other three arms. BrdU^+ ^cells were detected at 12 weeks post-implantation. Data suggested that NSCs embedded in HA-collagen biomaterial could facilitate re-innervations of damaged facial nerve and the artificial conduit of NSCs might offer a potential treatment modality to peripheral nerve injuries.

## Background

With the advent of surgical techniques and instruments, micro-sutures have considerably improved the management of peripheral nerve injuries. Autograft of the epineurium of an intact nerve remains to be the gold standard to bridge a nerve gap defect for the peripheral nerve lesion [23]. However, there are some limitations of the autologous nerve grafting technique including the limited number of donor nerves available, unaesthetic scaring, wound infection, wound pain, relatively long surgical time and learning curve for the success of nerve grafts, and poor regeneration. Controversial results were also reported on multiple anastomoses and acellular muscle grafts for cable grafting of large nerve defects [6,7,10]. Recent pre-clinical and clinical studies showed that allograft could be an alternative nerve graft [2,7,21]. Nerve allograft may act as a temporary scaffold across which host axons regenerate.

Natural or synthetic nerve guides were being developed and employed as alternatives to autografts in bridging nerve gap defects [9,22,24]. It was suggested that these scaffolds help direct axonal sprouting from the injured nerve and provide a conduit for diffusion of neurotrophic and neuroprotective factors produced by the lesioned nerve stumps [14]. An ideal scaffold should be biodegradable, biocompatible, non-toxic and mediate no immune response. In general, these biomaterials yielded poor results in the regeneration process of peripheral nerve injury [9,22]. Severe scarring and fibrosis are the most frequent problems.

Hyaluronic acid (HA) and collagen are ubiquitous and are major components of extracellular matrix (ECM) in the mammalian body. HA has a high capacity for holding water and possesses a high visco-elasticity. It adheres poorly to cells and prevents scarring. HA was noted to elicit positive biological effects on cells *ex-vivo*. Collagen is the main structural protein of connective tissues, and has great tensile strength and elasticity, and is employed in the construction of artificial skin substitutes. Components of ECM in tissue engineering have been actively studied. HA-collagen composite scaffolds were widely investigated recently for possible use as a biomaterial in tissue engineering scaffolds [26].

Stem cells are unspecified cells that can replicate, and under specific conditions, differentiate into various specialized cell types. NSCs transplantation was noted to promote functional recovery in animal models [4,15,17]. A recent study showed that *in vitro *culture of NSCs in three-dimensional HA-collagen matrix enhanced the differentiation of NSCs into neurons, astrocytes and oligodendrocytes [3]. However, the combinatorial effects of NSCs and HA-collagen composite scaffold in peripheral nerve repair are largely unclear. In this study, we made use of HA-collagen composite scaffold, NSCs and NT-3 as a nerve guide, effecter cells and neurotrophic/neuroprotective factor, respectively, and implanted the conduit of NSCs-implanted NT-3-supplemetned HA-collagen composite scaffold onto rabbits having induced peripheral nerve gap defect and evaluated the therapeutic effects on peripheral nerve lesion.

## Materials and methods

### Preparation of HA-Collagen composite conduit

Fresh solutions of 1% HA (Freda Biochemicals, Shandong, China) and 1% collagen (Sigma-Aldrich, St. Louis, MO) were mixed for six hours and were injected into the collagen conduit (Institute of Medical Equipment, Academy of Military Medical Sciences, China) which was tied at one end. The assembly was immersed in a solution containing the cross-linker, 1-ethyl-3-dimethylamino carbodiimide (EDC; Sigma-Aldrich) in 95% ethanol for 12 hours at 4°C. The cross-linked conduit was washed thrice in de-ionized water and freeze-dried at -20°C. The cross-linked matrices were then morphologically examined using scanning electron microscopy (JSM-6460LV) at 10 kV before and after release to down-streamed analyses.

### Cultures of NSCs

NSCs harvested from the neural cortex of E16 Sprague-Dawley rat embryos. For each rat, the head was decapitated and the whole brain was removed from the skull. Meninges, choroid plexus and coherent blood vessels were carefully stripped off. The brain tissue was cut into small pieces, triturated with a glass pipette and allowed to pass through a 28-mesh copper sieve to get rid of large chunks. Having washed thrice with Dulbecco's modified Eagle's medium (DMEM; Sigma-Aldrich), cells were seeded in 12 ml of high-glucose DMEM/F12 (Sigma-Aldrich) supplemented with 12.5 ng/ml basic fibroblast growth factor (FGF; Sigma-Aldrich) and 20 ng/ml epidermal growth factor (EGF; Sigma-Aldrich) onto a 75 cm^2 ^non-adherent tissue culture flask (Corning BV Life Sciences, Schiphol-Rijk, Netherlands) and maintained at 37°C in a humidified 5% CO_2_-incubator. Half of the spent medium was discarded and replenished with fresh culture medium every three days. Neurosphere cultures were passaged once a week by enzymatic segregation, using 0.25% trypsin and triturating with a glass pipette, and sub-cultured.

### Characterization of NSCs

Trypsinized cells with and without 10 μM bromodeoxyuridine (BrdU; Roche, Basel, Switzerland) labeling were allowed to grow on poly-L-ornithine- (Sigma) and laminin- (Sigma) coated coverslips. They were fixed in 4% paraformaldehyde (Sigma) for 20 minutes. Cells were permeabilized for five minutes with 0.3% Triton X-100 (Sigma) in phosphate-buffered saline (PBS) and then rinsed thrice with PBS. Non-specific binding was blocked with 10% normal goat serum (NGS; Zhongshanjinqiao, China) in PBS for 10 minutes. Cells were washed with 1% NGS in PBS and incubated overnight at 4°C with the following primary antibodies diluted in PBS containing 1% NGS: mouse IgG1 anti-class III β-tubulin (TuJ-III, 1:1,000; Exbio, Prahy, Czech), mouse IgG1 anti-glial fibrillary acidic protein (GFAP; 1:50; Santa Cruz Biotechnology, Santa Cruz, CA), and rabbit polyclonal IgG anti-galactocerebroside (GalC, 1:100; Santa Cruz). Labeled cells were detected with mouse IgG1 anti-BrdU (1:100; Millipore, Billerica, MA). After thrice washes with PBS, cells were incubated for 30 minutes with the corresponding secondary antibody: TRITC-conjugated goat anti-mouse IgG (1:100; Invitrogen, Carlsbad, CA), FITC-conjugated goat anti-mouse IgG (1:100; Santa Cruz) or FITC-conjugated goat anti-rabbit antibody (1:100; Santa Cruz). Washed cells without BrdU labeling were counter-stained with, either propidium iodide (PI; Sigma) or Hoechst 33342 (Invitrogen), and visualized using an inverted fluorescence microscope. Cells without primary antibody incubation were processed in the same manner as controls of false-positivity.

### Preparation of NSC for transplant

Neurospheres at passage three were labelled with 10 μM BrdU in the supplemented culture medium a day prior to nerve fiber transection to rabbits for *in vivo *study. BrdU-labeled cells were then trypsinized and washed thrice with PBS. Discrete NSCs were adjusted to 4 × 10^6^/ml in DMEM/F12 supplemented with 10 ng/ml neurotrophin-3 (NT-3; Sigma) for embedding to HA-collagen composite conduit.

### Embedding NSCs to HA-collagen conduit

Freeze-dried HA-collagen conduits were decontaminated by exposure to ultraviolet irradiation for an hour. NSCs (4 × 106) in one millilitre NT-3-supplemented DMEM/F12 culture medium were injected into the HA-collagen composite conduit of 7 mm in length. The NSC-embedded HA-collagen composite scaffold was then dipped into DMEM/F12 culture medium and incubated in 5% CO2-incubator at 37°C for two to three days.

### Induction of facial nerve injury and reconstruction to rabbits

Animal treatments were carried out to minimize pain or discomfort in accordance with the current protocols approved by the Institutional Animal Research Ethics Committee. A cohort of 39 normal adult New Zealand rabbits of 2.0 – 2.5 kg body weight was recruited for the study. They were allowed to gain access to food and water *ad libitum *in isolator cages at 25°C under a 12-hour light-dark cycle. Animals were randomly assigned into six groups: normal control (n = 5); bilateral facial nerve transected without reconstruction (n = 2); lateral nerve transected with implantation of HA-collagen composite scaffold (n = 7); lateral nerve transected with implantation of NSC and HA-collagen scaffold (n = 8); lateral nerve transected with implantation NT-3-supplemented HA-collagen scaffold (n = 6) and lateral nerve transected with implantation of NSC-embedded NT-3-supplemented HA-collagen composite scaffold (n = 11).

Having anesthetized by intravenous injection of 39 mg/kg sodium phenobarbital, rabbits were operated in a sterile condition. A horizontal incision was made to expose the main stem of the facial nerve. A segment of 2 mm was removed. A nerve gap defect of approximately 5 mm was apparent after contraction. A conduit of 7 mm in length was implanted onto the defect. Both nerve ends were sutured to the epineurium of the facial nerve using 10-0 nylon stitch. The skin incision was sutured. Animals were reared in isolator cages without any immunosuppressive prophylaxis.

### Behavioural assessment

#### 1. Ethology

Ethological methods were used to observe, record, and analyze animal behaviour in terms of signs and extents of muscular atrophy of lips, blink reflex, and ear motion of animals before and 12 weeks after peripheral nerve transection.

#### 2. Electromyography

Physiologic properties of lip muscles at rest and while contracting were evaluated and recorded using an electromyograph (Nicolet Viking IV, Portsmouth, VA). Electromyography in terms of time-latency, current threshold and voltage amplitude to a stimulus was performed on animals before and one, four, eight and 12 weeks after peripheral nerve transaction to assess the neuromuscular function. Pre-operated parameters were reckoned to be the reference values.

### Tissue processing for light and electron microscopy

Upon completion of *in vivo *monitoring, rabbits were anesthetized using sodium phenobarbital and euthanized. Blocks of facial muscles were fixed for three days in 4% paraformaldehyde and embedded in paraffin. Sections were de-waxed and stained with haematoxylin and eosin for histological examination. Toluidine blue staining was performed to assess regeneration [18]. Morphometric analyses were conducted to enumerate the fiber number, myelin sheath thickness, axon area and nerve fiber circumference using the Leica image analysis system (Leica Image Analyzer, Wetzlar, Germany).

Blocks of facial muscles and HA-collagen composite scaffolds were fixed with the modified Karnovsky's fixative containing 2% paraformaldehye (Sigma) and 2% glutaraldehyde (Sigma) in 0.1 M phosphate buffer for an hour and 1% osmium tetroxide (Sigma) in 0.1 M phosphate buffer for an hour. After rinsing with PBS for 15 minutes, specimens were dehydrated in a series of up-graded ethanol (70% to absolute) and further dried using hexamethyldisilazane (Sigma). Ultrathin sections were stained with uranyl acetate and lead citrate and mounted on aluminum stubs for electron microscopy.

### Immunohistochemistry

Immunohistochemical staining of BrdU and S-100 was performed to track the homing of NSC and to mark nerve fibers in the facial muscles of injured rabbits with and without reconstruction. Paraffin-embedded muscle sections of 5 μm in thickness were de-waxed and treated with 1 M hydrochloric acid to retrieve antigen of tissue sections that were masked by fixation. Endogenous peroxidase in muscle sections was denatured using 3% hydrogen peroxide. Upon completion of thrice washing in 0.01 M PBS for five minutes, sections were blocked with 5% normal goat serum in PBS for 30 minutes to suppress non-specific binding.

Primary streptavidin-conjugated antibodies, anti-BrdU (1:500; Sigma) and anti-S-100 (1:500; Sigma), were employed. Incubation was conducted at 37°C for 72 hours. After three washes in PBS, sections were incubated in biotin (1:300; Sigma) at room temperature for two hours. Sections were washed thrice with PBS and incubated with horseradish peroxidase-conjugated anti-biotin (1:300, Sigma) for three hours. Diaminobenzidine tetrahydrochloride substrate solution (Zhongshanjinqiao, China) was added for color development after three washes in PBS. Having been rinsed in gently running tap water, sections were counterstained with haematoxylin, dehydrated, cleared and mounted for visualization.

### Statistics analysis

Means and standard error of the mean (SEM) were calculated. The one-way analysis of variance (ANOVA) was applied to analyze continuous variables: time latency, threshold and amplitude of electromyogram and number, thickness, circumference and area of myelinated nerve fibers derived from rabbits with and without facial nerve injury and repair using NSC-embedded NT-3-supplemented HA-collagen composite scaffold to bridge the nerve gap. Differences between groups were regarded as significant if p ≤ 0.05.

## Results

### NSCs characterization

**Figure 1:**
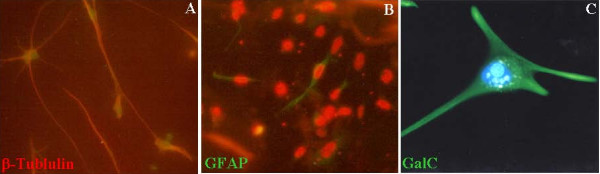
**Immunofluorescence staining of β-tubulin, glial fibrillary acidic protein (GFAP) and galactocerebroside (GalC) in neurosphere-derived cells cultured on poly-L-ornithine- and laminin-coated coverslips displaying a microglial morphology.** A: Cells with BrdU-labeled nuclei (green fluorescence) expressing β-tubulin (red fluorescence). B: Cells with propidium iodide-counterstained nucleus (red fluorescence) expressing GFAP (green fluorescence) and C: GalC (green fluorescence)-expressing cell counterstained with Hoechst 333442 (blue fluorescence).

Cells, which were derived from neurospheres and were allowed to grow on poly-L-ornithine- (Sigma) and laminin- (Sigma) coated coverslips, displayed a microglial morphology with protruding processes. Immunofluorescence staining of β-tubulin, GFAP and GalC demonstrated positive expressions in a substantial number of cells, suggesting that neurosphere-derived cells were able to differentiate into neuronal, astrocytic and oligodendrocytic progenies (Figure 1).

### NSCs growth on HA-collagen scaffold

**Figure 2:**
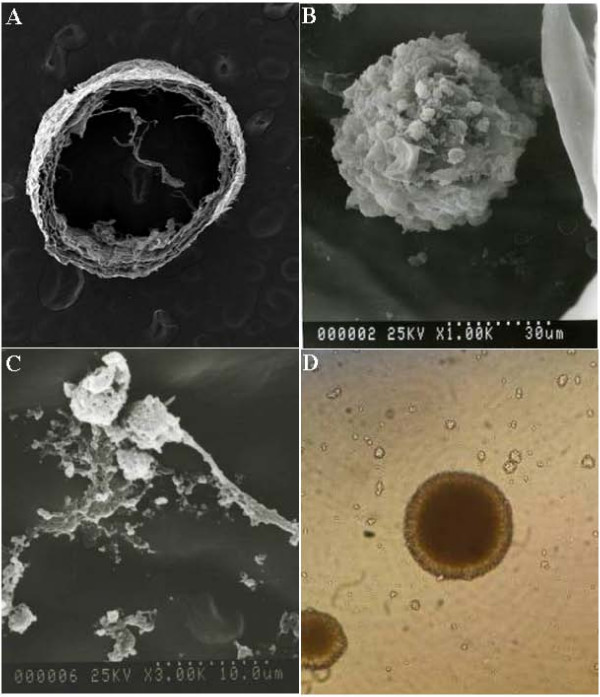
**Representative image of scanning electron microscopy of neural stem cell (NSC) at passage three derived from the neural cortex of E16 Sprague-Dawley rat embryos implanted on neurotrophon-3-supplemented hyaluronic acid (HA)-collagen composite scaffold and light microscopy of NSC in culture.** A: HA-collagen scaffold showing a conduit morphology with high porosity and surface area (1,000× magnification). B: The adhesion of a cell with spherical morphology and multiple short villi the scaffold after 24 hour culture (1,000× magnification). C: Cells with processes and protrusions adhered to the scaffold after culture for 48 hours (3,000× magnification). D: Cells segregated and formed neurospheres in culture without scaffold after 48 hours (400× magnification).

NSCs injected into NT-3-supplemented HA-collagen conduits were noted to adhere to the scaffolds and tended to differentiate, after being cultured for 24 and 48 hours, respectively (Figure 2). Protruding processes and neurite outgrowth were evident, compared to that of floating neurospheres.

### Recovery of animals

Recovery of the animals includes 2 index: ethology which is a measurement of animals' behaviour and electromyography which is used to measure neuromuscular function. According to the 2 index, the trend of recovery for the various groups is different.

#### 1. Ethology

All rabbits were noted to have normal lip muscles, blink reflex and ear motion prior to the facial nerve transaction. On 12 weeks post-surgery, rabbits with facial nerve transection displayed a muscular atrophy of upper lip and no erection and movement of the ear. Besides, there was no blink reflex. Injured rabbits implanted with HA-collagen scaffold (n = 7), or NSC and HA-collagen scaffold (n = 8), or NT-3-supplemented HA-collagen scaffold (n = 6), presented atrophic muscles of upper lip, torpid blink reflex and ear palsy. Injured rabbits with implantation of NSC-embedded NT-3-supplemented HA-collagen composite scaffold (n = 11) demonstrated slight blink reflex and ear movement but no erection. Muscular atrophy of upper lip was evident.

#### 2. Electromyography

Electromyography is a measurement of neuromuscular function. The prolongation of time-latency, increase of current threshold and decrease of voltage amplitude to stimuli may be attributed to an impairment of neuromuscular function after injury. When injury is recovering, shrink of time-latency and threshold, increase of amplitude is proposed to be observed. The mean ± SEM time latency of the study cohort of 39 rabbits before micro-surgery was 1.67 ± 0.30 ms, comparable to 1.68 ± 0.16 ms shortly after micro-surgery. Additional file 1 shows the time latency of rabbits with and without nerve fiber defect and scaffold implant at different time points. Minimal currents to elicit a visually detectable response in the animal cohort were depicted in Additional file 2. An increase of current threshold to stimulate nerves was evident. Rabbits which had facial nerve fiber defect, with and without scaffold implantation, exhibited higher current thresholds over 12 weeks of monitoring, compared to those derived from the normal counterparts. Thresholds shot up to the apexes on week four post-surgery, which were significantly higher than those derived from the normal control animals (p < 0.05), and declined gradually over 12 weeks. Rabbits which were untreated for facial nerve defect experienced the highest thresholds over 12 weeks post injury among their counterparts having implanted with different scaffolds. Readouts were correlated to the ethological assessments of animals displayed neuromuscular defects attributed to the atrophy of the upper lip and impairment of erection and movement of ipsilateral ears.

Current thresholds derived from rabbits implanted with HA-collagen scaffold (n = 7), NSC and HA-collagen scaffold (n = 8), and NT-3-supplemented HA-collagen scaffold (n = 6) over 12 weeks were comparable. On week 12, thresholds were still significantly higher than those before transection. In the arm of rabbits having implanted with NSC-embedded NT-3-supplemented HA-collagen composite scaffold for nerve fiber transection, the mean threshold on week eight was significantly less than that on week one (6.06 mA vs. 7.08 mA), though statistically higher than that derived from the normal controls (6.06 mA vs. 3.41 mA). On week 12, the mean threshold was comparable to that derived from the normal controls (4.11 mA vs. 3.41 mA). In concordance with the ethological observation, the animals were noted to have slight blink reflex and ear movement but no erection. A muscular atrophy of upper lip was still evident. Data suggested that acute facial palsy rested on the capacity of segmental nerve fibers to propagate a stimulus, albeit at a higher threshold, than that of normal fibers, and the rate and extent of regeneration. NSCs-embedded NT-3-supplemented HA-collagen composite scaffold was effective to enhance nerve fiber regeneration.

The amplitude of action potential derived from electromyography is the reflection of the neuromuscular response. Additional file 3 shows that voltage amplitudes derived from rabbits with facial nerve defect over 12 weeks decreased significantly, compared to that of rabbits before surgery (p < 0.05), attesting persistent facial nerve fiber defect.

### Regeneration of facial nerve

Light and Electron microscope, morphometric analysis and immunohistochemistry were used to examine regeneration of injured nerve.

#### 1. Light microscope

**Figure 3:**
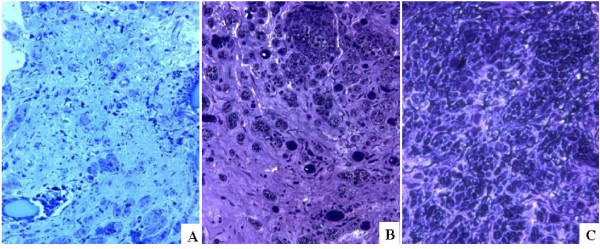
**Representative images of toluidine blue-stained tissue sections.** Nuclei and cytoplasm were stained bluish-purple and light blue, respectively. A: Connective tissue with unremarkable feature of rabbits undergone facial nerve fiber transection for 12 weeks (400× magnification). B: Sporadic clustering of nerve fibers and fascicles of various sizes developed in tissues of rabbits with facial nerve fiber transection and implant of NSC and HA-collagen scaffold for 12 weeks (400× magnification). C: An array of fascicles of relatively uniform size in tissues of rabbit after facial nerve fiber transection and implantation of NSC-embedded NT-3-supplemented HA-collagen composite scaffold for 12 weeks (400× magnification).

Light microscope observation of toluidine blue stained tissue sections revealed a significant dysplasia of myelinated nerve fibers in the lesioned tissue of rabbits after 12 weeks of nerve fiber transaction (Figure 3A). There was no infiltration of macrophages to the site of implant of NSC-embedded NT-3-supplemented HA-collagen composite scaffold or NSC and HA-collagen scaffold (data not shown), suggesting that the xenograft in conduit may be non- inflammatory, non-antigenic and immunologically tolerated by the recipient, without any sign of graft rejection, over 12 weeks of monitoring. Fascicles of various sizes and disorganized nerve fibers were noted to develop in rabbits having implant of HA-collagen scaffold, NSC and HA-collagen scaffold, and NT-3-supplemented HA-collagen composite scaffold (Figure 3B). Fascicles and nerve fibers were more remarkable and organized in rabbits having NSC-embedded NT-3-supplemented HA-collagen composite scaffold (Figure 3C), which resembled to that of normal rabbit tissues (data not shown). However, the degeneration was explicit.

#### 2. Morphometric analysis

The extents of nerve fiber regeneration in the cohort of rabbits implanted with different scaffold assemblies were assayed in the term of the absolute number, thickness, circumference and area at the proximal and distal nerve stumps 12 weeks after surgery (Additional file 4). Rabbits which had no management of nerve fiber damage were not enrolled to the assessment as there was little regeneration. The mean number of myelinated nerve fibers derived from rabbits having undergone implantation of NSC-embedded NT-3-supplemented HA-collagen composite scaffold was comparable to that of normal control (p < 0.05). The mean areas and circumferences of myelinated nerve fibers derived from rabbits having implanted NSC and HA-collagen scaffold, and NSC-embedded NT-3-supplemented HA-collagen scaffold, were similar to those of normal controls (area and circumference; p < 0.05). Data suggested that NSC in conjunction with HA-collagen composite scaffold can enhance nerve fiber regeneration.

Distinct thinning of the myelin sheath was noted in the two arms of rabbits with HA-collagen scaffold and NT-3-supplemented HA-collagen, respectively, compared to that of the normal control (p < 0.05). Conversely, the mean values of myelin sheath thickness of nerve fibers in tissue sections of normal rabbits and rabbits implanted with NSC-embedded NT-3-supplemented HA-collagen composite scaffold were comparable. The myelin sheath of rabbits receiving NSC and HA-collagen scaffold was noted even thicker (Additional file 4). It suggests that NSC and HA-collagen composite graft is effective in alleviating the extent of degeneration mediated by facial nerve fiber defect.

#### 3. Electron microscope

Scanning electron microscopy showed NSC adhered to the scaffold in 24 hours. Long axons were noted after culturing for three days. Transmission electron microscopy demonstrated intact myelin sheath, microfilament and microtubule in nerve fibers of tissue sections of normal control rabbits. In line with light microscopy, transmission electron microscopy illustrated the prevalence of connective tissues and hyperplasia of blood vessels in rabbits without management of facial nerve fiber defect. Myelinated nerve fibers were sporadically encountered in tissue sections of rabbits implanted with HA-collagen scaffold, NSC and HA-collagen scaffold, and NT-3-supplemented HA-collagen scaffold. Degeneration was evident. Observation by the light microscope also helped in the observation of similar phenomena. A thickening of myelin sheath was noted in the arm of rabbits having grafted with NSC and HA-collagen scaffold (data not shown). In rabbits with implant of NSC-embedded NT-3-supplemented HA-collagen composite scaffold, the alignment of myelinated nerve fibers resembled to that of normal control, however degeneration was explicit.

#### 4. Immunohistochemistry

**Figure 4:**
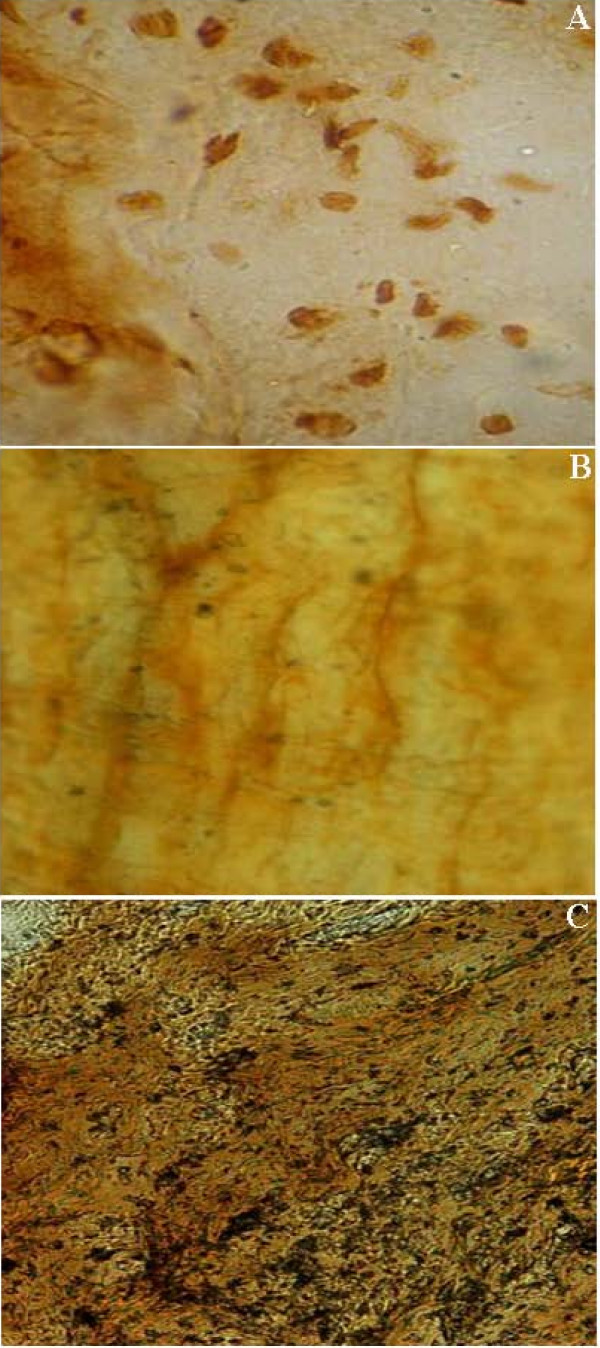
**Representative images of immunohistochemical staining of BrdU and S-100.** A: Localization of darkly brownish stained BrdU^+ ^cells to transected tissues of rabbits having a segment of facial nerve fiber removed and implanted with NSC and HA-collagen scaffold, or NSC-embedded NT-3-supplemented HA-collagen composite scaffold, for 12 weeks (800× magnification). B: Brownish stained S-100+ facial nerve fibers in regular waves in normal tissue section of rabbits. No hyperplasia was detected (400× magnification). C: Waves of S-100^+ ^nerve fibers in a less organized manner and hyperplasia of connective tissue were noted in tissues of rabbits after facial nerve fiber transection and implantation of NSC-embedded NT-3-supplemented HA-collagen composite scaffold for 12 weeks (400× magnification).

Immunohistochemical staining demonstrated BrdU^+ ^cells in tissues of rabbits implanted with NSC together with HA-collagen scaffold, and NSC-embedded NT-3-supplemented HA-collagen composite scaffold suggesting implanted cells could survive for at least 12 weeks. (Figure 4A). It was notable that the donor cells migrated and homed to lesioned junctions of transected tissues. S-100 staining revealed regular waves of nerve fibers in normal facial muscles of control rabbits with normal plasticity (Figure 4B), which were in contrast to the predominance of connective tissues and apparent angiogenesis in a chaotic manner in rabbits without management of nerve fiber truncation (data not shown). A lesser degree of angiogenesis and a few irregularly aligned nerve fibers were noted in three arms of rabbits implanted with HA-collagen scaffold, NT-3-supplemented HA-collagen scaffold, or NSC and HA-collagen scaffold. Figure 4C illustrated waves of nerve fibers, though less organized and hypertrophic tissues from rabbits implanted with NSC-embedded NT-3-supplemented HA-collagen composite scaffold for nerve fiber damage.

## Discussion

Injury to peripheral nerves presents a challenge to the recovery of nerve function. Despite nerve auto-graft remaining as a widely practiced micro-surgical technique for peripheral nerve defect, NSCs therapy and nerve grafting of synthetic conduit made up of biomaterials may be potential modalities for repair. In this study we managed peripheral nerve injury by grafting NSCs-embedded NT-3-supplemented HA-collagen composite scaffold to bridge facial nerve fiber gaps in rabbit models. Donor cells were noted to home to lesioned areas. Tissue regeneration was evident with a remarkable development of fascicles and nerve fibers. Degeneration was reduced as shown by apparently normal thickness of myelin sheath of nerve fibers. Ethology could not display any significant neuromuscular recovery.

Pertaining to the super biocompatibility, hydrophilic activity, non-immunogenic property, biodegradability and inertness in mediating scarring and fibrosis, synthetic biomaterials have drawn much attention in tissue reconstruction and regeneration research [3,27]. HA was noted to play a supporting role for developmentally immature neural cells *in vivo *[25]. HA matrix was also shown to induce neurite outgrowth without glial scar development *in vivo *[13]. Cell differentiation and synapse formation was evident in *ex vivo *studies of NSC in three-dimensional collagen gels [20]. The architecture of HA-collagen composite scaffold provides a conduit of high surface area and porosity for cell adhesion and guide for the nerve fibers [26]. Not only it is requisite to nerve regeneration, but also it is vital to accommodate effecter molecules and cells. Various signals and neural factors were incorporated into the conduit to minimize infiltration of fibrous tissue and enhance neurite outgrowth [13].

In the study it was noted that the conjunct nerve was embedded with connective tissues 12 weeks after implantation. There were neither signs of inflammation, accretion nor destruction. When dissected, no remnants of the composite scaffold were noted. Readouts suggested that the HA-collagen scaffold was biocompatible and biodegradable. The clearance rate of the scaffold was primarily in phase with that of regeneration.

The potential of signalling molecules, inducing factors, cytokines, or effecter cells embedded in synthetic composite scaffolds for tissue regeneration, especially in the treatment of peripheral nervous system injuries and defects, has drawn much interest. NT-3 which is a neurotrophic factor in the nerve growth factor family of neurotrophins helps support the survival, growth and differentiation of both existing and new neurons and synapses *in vivo *and *ex vivo*. In the study the supplement of NT-3 to NSCs embedded in HA-collagen composite scaffold not only enhanced NSCs differentiation and neurite outgrowth, but also provided growth factor to promote endogenous regeneration and lessen degeneration.

Peripheral nerve regeneration was evident with the implantation of conduits pre-seeded with Schwann cells which secrete neurotropic and neuroprotective factors and re-myelinate defect nerve [5]. NSCs, which are able to differentiate *ex vivo *into neurons, astrocytes and oligodendrocytes and express constitutively neurotropic and neuroprotective factors, were reported to promote extensive host axonal growth after spinal cord injury [1,8,19].

The fate of implanted NSCs was noted to be dictated by the *in vivo *micro-environment [16]. The reactive niche might induce NSC into progenitors and effecter cells of the neural lineage that would enhance regeneration and alleviate degeneration. Besides, low immunogenicity and antigenicity are the fortes of NSCs. It was reported that allogeneic NSC survived at least four weeks in a non-immune-privileged site, during which they neither sensitized their hosts nor expressed detectable levels of major histocompatibility complex class I or II, suggesting that NSCs lack immunogenicity and resist rejection [11,12]. In this study, although rat NSCs were used as implanted cells to rabbits and no immunosuppressant was used, no evident immunal rejection was observed. This is consistent with previous reports, even if more solid evidences and proof are still needed.

Readouts of the ethology, electromyography, light microscopy, morphometric analysis, immunohistochemistry and transmission electron microscopy suggested that animals, which had no treatment for peripheral nerve injury, displayed an extremely limited auto-regeneration. The conduit provided guides to the regenerating nerve fibers. Despite the results derived from morphometric analyses were not totally in line with those of electromyography, the degree of regeneration from animals with peripheral nerve defect and implanted with NSC-embedded NT-3-supplemented HA-collagen composite scaffold appeared to have the greatest extent of regeneration among all arms of injured animals. It might be attributable to the differentiation of NSC into effecter glial cells and oligodendrocytes participating in regeneration. However, more work is needed to test the hypothesis. NSC-derived neurotrophic and neuroprotective factors also have roles in this issue. Besides, HA-collagen composite scaffold offered a favourable platform for cell anchoring and trafficking, guiding axonal sprouting from nerve stumps, and re-innervations, not to mention nutrition conveyance.

The impaired transmission of neural impulses resulted from facial nerve fiber and axonal discontinuity. Minimal neuromuscular excitability in terms of current threshold and voltage amplitude was hampered shortly after peripheral nerve injury of animals with and without implant of nerve graft. Despite the current threshold of animals implanted with NSC-embedded NT-3-supplemented HA-collagen composite scaffold reached a comparatively normal level, the neuromuscular function displayed no significant improvement.

A hypertrophy of myelin sheath of nerve fibers was noted in animals implanted with NSC and HA-collagen scaffold for peripheral nerve fiber defect, which also appeared but was not so evident in injured animals having implant of NSC embedded NT-3-supplemented HA-collagen scaffold. Reasons were not clear. It is unknown whether NT-3 supplement or NSCs transplantation in arresting the thickening of myelin sheath in this setting. Conversely, electron microscope observation of the tissue sections of facial nerve defect animals, having undergone implant of NSC-embedded NT-3-supplemented HA-collagen composite scaffold, revealed that a number of nerve fibers were still un-myelinated. Degeneration and swelling of myelin lamellae was also evident. Data suggested that there is still room for improvement of the cell scaffold.

In conclusion, the *in vivo *study described an alternative to manage peripheral nerve defect and enhance regeneration by grafting NSC-embedded NT-3 supplemented HA-collagen composite scaffold to bridge the nerve gap. This highlights the importance of optimizing the cell scaffold for translational medicine.

## Competing interests

The authors declare that they have no competing interests.

## Authors' contributions

HZ and TWY collected and analyzed data. KST interpreted data and wrote the manuscript. CRS, JL and HH acquired data. FZC analyzed and interpret data. YHA designed the study and approved the manuscript.

## Supplementary Material

Additional file 1 **The time latency between distal stimulation and recording of electromyography of rabbits before and after facial nerve transection with and without implant of scaffold for repair.** The programme required to open this file is ACDSee
Click here for file

Additional file 2 **The current threshold of electromyography of rabbits before and after facial nerve transection with and without implant of scaffold for repair.** The programme required to open this file is ACDSee
Click here for file

Additional file 3 **The voltage amplitude of electromyography of rabbits before and after facial nerve transection with and without implant of scaffold for repair.** The programme required to open this file is ACDSee
Click here for file

Additional file 4 **Morphometric analysis of peripheral nerve regeneration.** Click here for file
