# Supplementary figures and images for: Implantation of neural stem cells embedded in hyaluronic acid and collagen composite conduit promotes regeneration in a rabbit facial nerve injury model
Source: J Transl Med. 2008 Nov 5;6:67. doi: 10.1186/1479-5876-6-67 (PMC2614414; doi:10.1186/1479-5876-6-67)

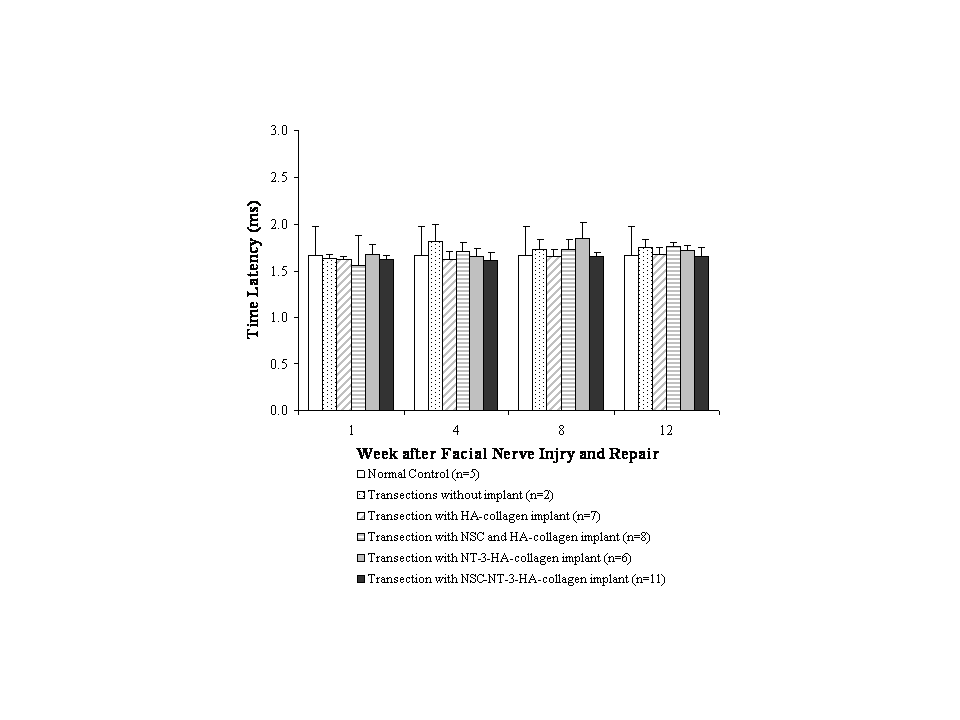

Supplement: Additional file 1 — The time latency between distal stimulation and recording of electromyography of rabbits before and after facial nerve transection with and without implant of scaffold for repair. The programme required to open this file is ACDSee [file 1479-5876-6-67-S1.tiff]

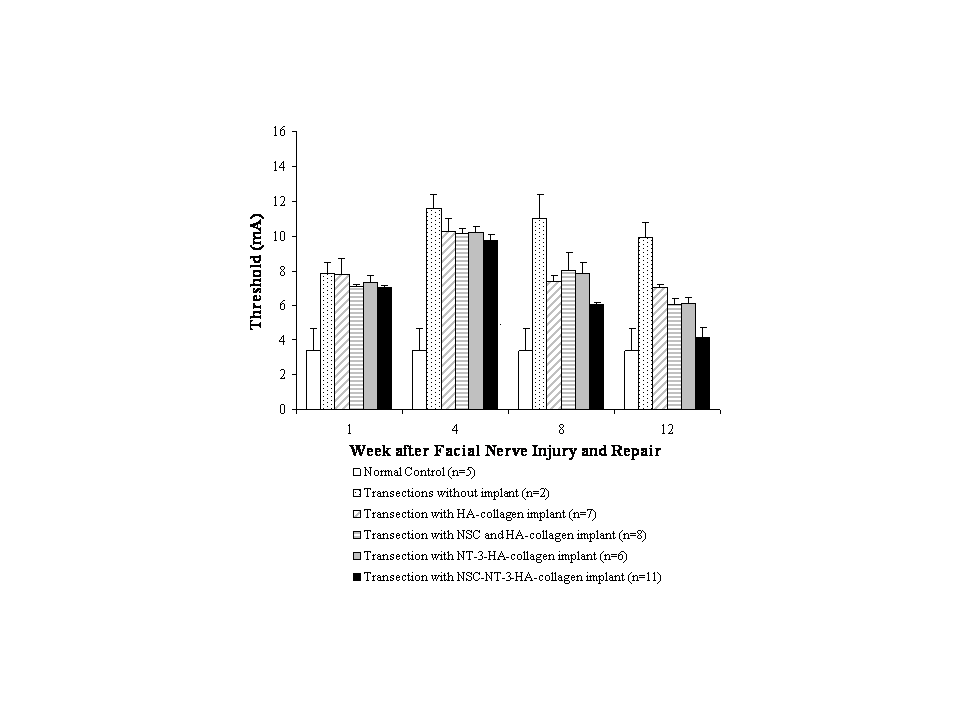

Supplement: Additional file 2 — The current threshold of electromyography of rabbits before and after facial nerve transection with and without implant of scaffold for repair. The programme required to open this file is ACDSee [file 1479-5876-6-67-S2.tiff]

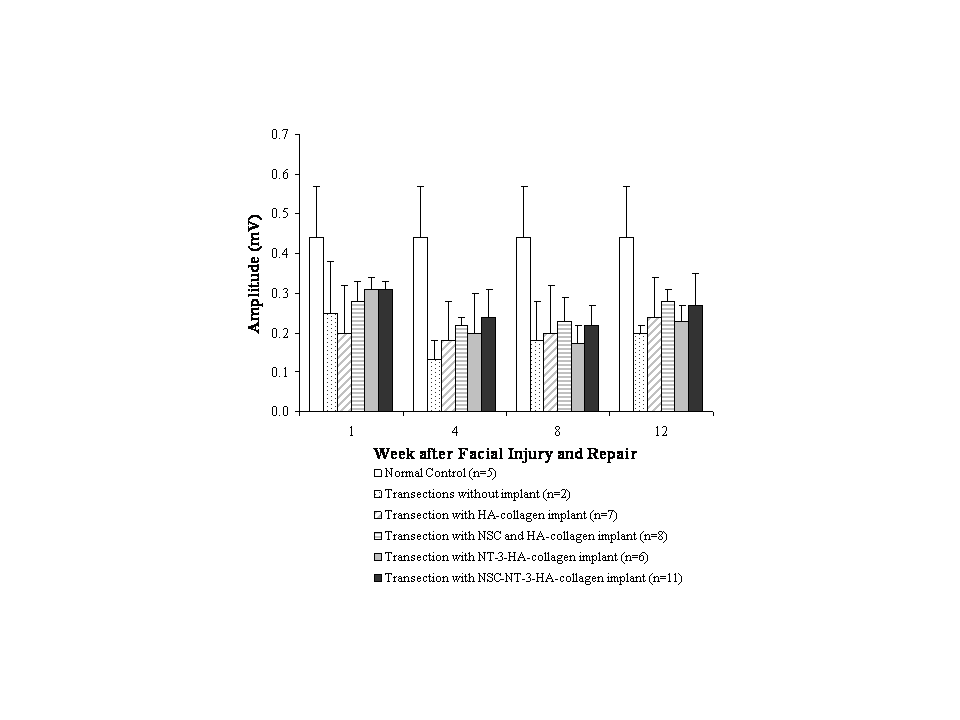

Supplement: Additional file 3 — The voltage amplitude of electromyography of rabbits before and after facial nerve transection with and without implant of scaffold for repair. The programme required to open this file is ACDSee [file 1479-5876-6-67-S3.tiff]
