# Supplementary material for: Implantation of neural stem cells embedded in hyaluronic acid and collagen composite conduit promotes regeneration in a rabbit facial nerve injury model
Source: J Transl Med. 2008 Nov 5;6:67. doi: 10.1186/1479-5876-6-67 (PMC2614414; doi:10.1186/1479-5876-6-67)
Supplement: Additional file 4 — Morphometric analysis of peripheral nerve regeneration. [file 1479-5876-6-67-S4.doc]

Table 1 Morphometric analysis of peripheral nerve regeneration

|  | Mean ± Standard Error of the Mean | | | |
| --- | --- | --- | --- | --- |
| Study arm of rabbits  with facial nerve fiber injury | Nerve Fiber Number | Myelin Sheath Thickness  (μm) | Axon  Area  (μm^2^) | Nerve Fiber Circumference  (μm) |
| Normal control without injury  (n=5) | 209.3±60.1 | 0.72±0.02 | 21.6±4.33 | 26.45±1.00 |
| HA-collagen scaffold  (n=7) | 52.3±49.6* | 0.17±0.30* | 9.95±7.23* | 11.94±2.67* |
| NSC and HA-collagen scaffold  (n=8) | 126.4±41.8* | 1.15±0.43* | 23.99±1.62 | 26.42±2.49 |
| Neurotrophin-3-supplemented HA-collagen scaffold  (n=6) | 102.3±48.6* | 0.45±0.33* | 19.99±13.54* | 19.17±13.01* |
| NSC-embedded NT-3-supplemented HA-collagen scaffold  (n=11) | 184.4±48.9 | 1.02±0.25 | 25.58±12.74 | 26.27±7.87 |

* p <0.05 compared to that of normal control
